# Hazardous alcohol use and associated factors in a rural Ethiopian district: a cross-sectional community survey

**DOI:** 10.1186/s12889-016-2911-6

**Published:** 2016-03-03

**Authors:** Solomon Teferra, Girmay Medhin, Medhin Selamu, Arvin Bhana, Charlotte Hanlon, Abebaw Fekadu

**Affiliations:** Department of Psychiatry, College of Health Sciences, Addis Ababa University, Addis Ababa, Ethiopia; Aklilu Lemma Institute of Pathobiology, Addis Ababa University, Addis Ababa, Ethiopia; Health Systems Research Unit, South African Medical Research Council, School of Applied Human Sciences, University of KwaZulu-Natal , Durban, South Africa; King’s College London, Institute of Psychiatry, Psychology and Neuroscience, Centre for Global Mental Health, London, UK; King’s College London, Institute of Psychiatry, Psychology and Neuroscience, Centre for Affective Disorders, London, UK

**Keywords:** Alcohol, Cross-sectional, Community survey, Hazardous use, Rural, Sub-Saharan Africa, Ethiopia

## Abstract

**Background:**

Alcohol related health and social problems are on the rise in sub-Saharan Africa. This survey reports the prevalence and associated factors for hazardous drinking in rural Sodo district, southern Ethiopia. The survey was part of a multi-center study, Programme for Improving Mental Health Care (PRIME), which is a consortium of research institutions and ministries of health of five low and middle income countries, namely Ethiopia, India, Nepal, South Africa and Uganda in partnership with UK institutions and World Health Organization (WHO).

**Methods:**

A cross-sectional community survey was conducted involving 1500 adults, age 18 and above, recruited using multi-stage random sampling. Data on alcohol use was collected using the Fast Alcohol Screening Test (FAST). Standardised instruments were used to measure potential associated factors, including a validated adaptation of the Kessler 10 (psychological distress), the List of Threatening Experiences (number of adverse life events). Exploratory multivariable logistic regression was conducted to examine factors associated with hazardous alcohol use.

**Results:**

The overall prevalence of hazardous alcohol use was found to be 21 %; 31 % in males and 10.4 % in females, *P* < 0.05. Factors independently associated with hazardous alcohol use were being male (adjusted OR = 4.0, 95 % CI = 2.44, 6.67), increasing age, having experienced one or more stressful life events (adjusted OR = 1.71, 95 % CI = 1.18, 2.48, and adjusted OR = 2.12, 95 % CI = 1.36, 3.32 for 1–2 and 3 or more adverse life events, respectively) and severe psychological distress (adjusted OR = 2.96, 95 % CI = 1.49, 5.89). High social support was found to be protective from hazardous alcohol use (adjusted OR = 0.41, 95 % CI = 0.23, 0.72).

**Conclusion:**

High level of hazardous alcohol use was detected in this predominantly rural Ethiopian setting. The finding informed the need to integrate services for hazardous alcohol use such as brief intervention at different levels of primary care services in the district. Public health interventions to reduce hazardous alcohol use also need to be launched.

## Background

Consumption of alcoholic beverages is a common practice among adults globally. Some of the consumption patterns may surpass acceptable limits and result in multitude of problems, including physical health, psychological and social problems. Around 4.9 % of the world’s adult population is believed to suffer from alcohol use disorder [1]. Besides the direct harm caused by alcohol such as liver injury, it is also an important risk factor for many chronic diseases, notably high blood pressure and other cardiovascular diseases related morbidity and mortality [2]. It is also an important risk factor for injury related morbidity and mortality. The World Health Organization (WHO) reports indicate that the global alcohol related mortality from cancer, liver cirrhosis and injury accounted for 2.8 % of all deaths, 1.3 % for women and 4.1 % for men globally [3].

Although there is a dearth of prevalence studies in Africa, the existing literature indicates that alcohol use disorders are common. Evidence from an upper middle-income country in sub-Saharan Africa, South Africa (SA), showed that 9 % of the population aged 15 years or older engaged in risky or hazardous or harmful drinking. More men had risky use than women, 17 % and 2.9 % respectively [4]. Another study done in a general outpatient population in different hospitals in SA involving 1,532 adults using Alcohol Use Disorder Identification Test (AUDIT) [5] found that 41.2 % of men and 18.3 % of women had hazardous drinking and 3.6 % of men and 1.4 % of women met criteria for probable alcohol dependence or harmful drinking as defined by AUDIT [6]. In another study in SA that looked at the burden of disease attributable to alcohol in 2000, 7.1 % of all deaths in SA were attributed to alcohol. It also accounted for 7 % of the total disability adjusted life years (DALYs). The two most important causes of alcohol related death were injury and cardiovascular incidents [7].

According to the 2011 Demographic and Health Survey (DHS) report by Central Statistics Authority (CSA) of Ethiopia, a national survey involving a representative sample from the age group 15–49 year old, 45 % of women and 53 % of men reported a history of alcohol consumption in their life time, 48 % of women and 53 % men of ever drinkers reported consumption of alcohol six or more days in the past month. Consumption rate was higher in urban areas, and the rates increased with age for both men and women [8].

Homemade alcoholic beverages include *tella* (local beer with alcohol content 2-4 %), *tej* (honey wine with alcohol content 7-11 %) and *araqe* (strong distilled liquor with alcohol content up to 45 %) [9], these beverages are consumed at home or in small traditional bars, *‘tella bet’* and *‘araqe bet’* meaning *tella* house and *araqe* house, where rural farmers and people in the lower socioeconomic status go to satisfy their needs. Most people consume these alcoholic beverages during traditional ceremonies, holidays or while taking respite from farm activities, but it is also common to consume these beverages during market days. Farmers who go to the market to sell their goods will normally end up drinking in the small bars which sell alcoholic beverages [9].

Prevalence Studies done in different parts of Ethiopia using the four-item “CAGE” screening questionnaire [10] have shown that hazardous alcohol drinking was 3.7 % and 2.7 % in rural and urban areas respectively [11, 12]. In another study carried out in a semi-nomadic population in Southern Ethiopia using a structured diagnostic interview, the Composite International Diagnostic Interview (CIDI), the prevalence rate of alcohol dependence was 1.6 %, more in men than women, 3.7 % and 0.1 % respectively [13]. Alcohol consumption has also been found to be associated with risky sexual behaviors and injuries in the Ethiopian context. For instance, a large study done in Addis Ababa involving in-school and out-of-school youth found significantly increased risky sexual behavior in youth who consumed alcohol and khat, an amphetamine-like stimulant [14].

This survey was part of a multi-center study, Programme for Improving Mental Health Care (PRIME), which is a consortium of research institutions and ministries of health of five low and middle income countries, namely India, Ethiopia, Nepal, South Africa and Uganda in partnership with UK institutions and World Health Organization (WHO) [15]. PRIME aims to generate evidence on approaches of integrating mental healthcare into primary care for selected priority disorders, including alcohol used disorders (AUD). In Ethiopia PRIME is being implemented in the Sodo District, a predominantly rural district in South Ethiopia. This study reports the prevalence of hazardous alcohol use and associated factors to inform the PRIME district level intervention.

## Methods

A cross-sectional survey of adults aged 18 years and above was conducted. Participants were selected randomly from all sub-districts (kebeles) of the Sodo district proportional with the population size of each sub-district.

### Participants and setting

Sodo district, a predominantly rural district, is located in the Gurage Zone, Southern Nations, Nationalities and Peoples Region (SNNPR), 100 km south of the capital of Ethiopia, Addis Ababa. The population of the district is 161,952 persons (79,356 men; 82,596 women) living in 58 sub-district [16]. Different ethnic groups live in the district: Sodo Gurage (85.3 %), Oromo (11.6 %) and Amhara (1.5 %) and others (1.6 %). The lingua franca, Amharic, is also the working language in the district. The majority follows the Coptic Orthodox Christian faith (97 %) and Muslims make up 2.3 %. The district has 62 health facilities, all at the level of primary healthcare: 8 public and 1 run in a public private partnership arrangement and more than 50 health posts in the community, all government owned, run by 2 female health extension workers who have a high school education plus one year training in preventive, promotive and very basic curative services. The health center in Bu’i, the district capital, is undergoing upgrading to get the status of a rural hospital. The nearest hospital is located in Butajira town, 30 km South of Bu’i town. Mental health care provision is being started in the district with the help of the PRIME project. Prior to this service being initiated by PRIME, people with mental and substance use disorders had to travel to Butajira hospital, which has a psychiatric nurse led outpatient service or go to the capital Addis Ababa to receive mental health services. PRIME was launched in Sodo because the district was believed to represent rural Ethiopia and is located close to the capital city where specialist mental health services exist and is linked to the Butajira Demographic Surveillance Site, DSS, which has research infrastructure [17, 18].

Inclusion criteria were being aged 18 years and above, able to give consent and residing in the particular sub-district for six or more months.

### Sampling method

Multistage sampling method was used. First, the number of households in each sub-district was determined. After samples of households were calculated from each sub-district, participants were selected proportionate to the number of households in each sub-district. Consequently, the number of adults included in the study was equal to the number of households selected in each sub-district. When there were more than one eligible adult in a household, the sample was selected using random sampling. The total sample size was 1,500, determined based on the assumption of prevalence of 10 % (a conservative estimate) and a design effect of 1.5 %, a precision of 0.02 % and a non response rate of 15 %.

### Screening for alcohol use disorder

Screening for alcohol use was carried out using the Fast Alcohol Screening Test (FAST), a short screening questionnaire for hazardous drinking comprising four questions which can be easily administered in a minute or less, derived from Alcohol Use Disorder Identification Test (AUDIT) [5, 19]. The items in FAST include the following questions: 1) MEN: How often do you have EIGHT or more drinks on one occasion? WOMEN: How often do you have SIX or more drinks on one occasion? (Score: 0 = Never, 1 = Less than Monthly, 2 = Monthly, 3 = Weekly, 4 = Daily or almost daily). 2) How often during the last year have you been unable to remember what happened the night before because you had been drinking? (Score: 0 = Never, 1 = Less than Monthly, 2 = Monthly, 3 = Weekly, 4 = Daily or almost daily). 3) How often during the last year have you failed to do what was normally expected of you because of drinking? (Score: 0 = Never, 1 = Less than Monthly, 2 = Monthly, 3 = Weekly, 3 = Daily or almost daily). 4) In the last year has a relative or friend, or a doctor or other health worker been concerned about your drinking or suggested you cut down? (Score: 0 = No, 2 = Yes, on one occasion, 4 = Yes, on more than one occasion). A score of 3 or more out of 16 indicates the occurrence of hazardous alcohol use [20, 21], a pattern of drinking which is associated with increased risk of adverse psychological or physical consequences in the future [21]. The FAST has been found to have better psychometric properties than the CAGE [22, 23], with sensitivity of 0.93 and specificity of 0.88 [20], and comparable to the AUDIT [24]. It is also reported to have a higher sensitivity and specificity than the AUDIT when used in emergency departments [24]. Although not validated in the Ethiopian setting, the AUDIT has been validated in neighbouring African countries [25, 26]. Local alcoholic beverages were converted into standard equivalent alcohol units when reported by the respondents using different containers such as flasks, cans and glasses. Estimates of the alcohol content of the different locally available beverages has been determined before and used to estimate the amount of alcohol units consumed [9].

Psychological distress was assessed using the Kessler Psychological Distress Scale (K-10) [27]. List of Threatening Experiences (LTE), with 12 categories of adverse life events, was used for assessing stressful life experiences in the past six months. Examples of items in the LTE include death of close persons, loss of relationships, imprisonment, and being the victim of theft [28]. The Oslo-3 Social Support Scale (OSS-3), a three item scale exploring the number of close confidants, perceived level of concern from others and perceived ease of getting help from neighbours, was used to assess social support [29].

### Administration of assessment instruments

The FAST alcohol screening questionnaire was part of the screening instruments compiled to collect information on a range of mental health and related issues in the PRIME Project in Sodo. Trained community health workers administered the questionnaire translated to the local language of Amharic. The supervisors of data collectors were nurses who had many years of experience working in mental health research.

### Data analysis

Data was entered using Epi-data version 3.1 and analyzed using SPSS-20 (IBM Corp 2012) respectively. Using descriptive methods, the data was summarized, prevalence of hazardous alcohol use was determined and odds ratios (OR) were obtained using logistic regression. The effect of Sociodemographic (age, sex, education, marital status, occupation, residence, ethnicity, and perceived relative wealth) and psychosocial factors (social support, life events, mental distress) on hazardous drinking was explored using crude and adjusted odds ratios.

### Ethical considerations

Ethical approval was obtained from the Institutional Review Board of the College of Health Sciences of Addis Ababa University. Participants gave their written informed consent after adequate information about the study, and the potential benefits and risks, had been provided.

## Results

### Socio-demographic characteristics

A total of 1,500 participants were interviewed, of whom 49.5 % were males. Ninety percent of the participants resided in rural areas. Farming was reported to be the occupation by 51.6 % of the participants, being housewife was the predominant occupation reported by the women and 8.7 % of the respondents reported to be engaged in private business. The majority belong to the Gurage Ethnic group (94.6 %). More than 90 % were aged below 60 years, and more than 75 % were married. Regarding literacy, 42.8 % were illiterate, 23.8 % able to read and write, 24.5 % primary education, only 8.8 % had secondary and above education. Close to 90 % described their income as average or low relative to others in their neighbourhood, taken as a crude estimate of wealth.

The overall prevalence of hazardous alcohol use was found to be 21 %, 31 % in males and 10.4 % in females, *P* < 0.05. Forty two percent of respondents reported experiencing one or more life events in the past six months. Similarly, nearly 41.9 % described their social support as poor, and only 11.7 % reported to have strong social support. Regarding the findings on psychological distress as measured by the Kessler-10 psychological distress scale, 13.9 %, 9.0 %, and 5.2 % scored mild, moderate and severe respectively.

Details of the socio-demographic and clinical characteristics are presented in Table 1.

**Table 1:** Socio-demographic and clinical characteristics of participants in Sodo, Southern Ethiopia

Characteristics		Number (%)
Sex	Male	742 (49.5)
	Female	758 (50.5)
Age group (years)	18-24	224 (15.1)
	25-34	425 (28.6)
	35-44	395 (26.6)
	45-49	322 (21.6)
	60 and above	122 (8.2)
Marital status	Single	260 (17.5)
	Married	1129 (75.8)
	Divorced/Widowed	100 (6.7)
Education	Illiterate	592 (42.9)
	Able to read	328 (23.8)
	Primary	338 (24.5)
	Secondary and above	122 (8.8)
Residence	Urban	139 (9.3)
	Rural	1352 (90.7)
Ethnicity	Gurage	1414 (94.6)
	Other	81 (5.4)
Occupation	Farmer	770 (51.6)
	Housewife	405 (27.1)
	Private business	130 (8.7)
	Daily laborer	75 (5.0)
	Other	113 (7.6)
Perceived relative wealth	Poor	499 (34.1)
	Average	796 (54.4)
	High	168 (11.5)
Hazardous alcohol use	Male	230 (31.8)
	Female	79 (10.4)
Number of adverse life events	No life event	769 (58.0)
	1-2 events	343 (25.9)
	≥3 events	214 (16.1)
Social support	Poor	581 (41.9)
	Moderate Support	644 (46.4)
	Strong support	162 (11.7)
Severity of psychological distress	No distress	1059 (71.9)
	Mild	204 (13.9)
	Moderate	133 (9.0)
	Severe	76 (5.2)

### Hazardous alcohol use and associated factors

Table 2 shows the result of logistic regression analysis of the different factors and their association with hazardous alcohol use; both crude and adjusted results were calculated and reported. Males had significantly increased risk of hazardous alcohol use both in the crude and adjusted analysis (adjusted OR = 4.0, 95 % CI = 2.44, 6.67, *P* < 0.05). All of the age groups 25 years or older had significantly higher rates of hazardous alcohol use in the crude analysis; whereas, difference was not significant for age group 60 and above in the adjusted analysis. Number of adverse life events was significantly associated with hazardous alcohol use both in crude and adjusted analysis (adjusted OR = 1.71, 95 % CI = 1.18, 2.48, *P* < 0.05 and adjusted OR = 2.12, 95 % CI = 1.36, 3.32, *P* < 0.05 for 1–2 and 3 or more adverse life events respectively). The other factors significantly associated with hazardous alcohol use was severe psychological distress (adjusted OR = 2.96, 95 % CI = 1.49, 5.89, *P* < 0.05). High social support was found to be protective from hazardous alcohol use in the adjusted analysis (crude OR = 0.64, 95 % CI = 0.40, 1.01, adjusted OR = 0.41, 95 % CI = 0.23, 0.72, *P* < 0.05). Marital status and level of literacy were not significantly associated with hazardous alcohol use. Similarly, place of resident, ethnicity, occupation and perceived relative wealth were not significantly associated with hazardous alcohol use.

**Table 2:** Sociodemographic and clinical factors associated with hazardous use of alcohol in Sodo, Southern Ethiopia

Characteristics		Crude OR	95 % CI	Adjusted OR	95 % CI	*P*-value*
Sex	Male	4.0	3.03-5.26	4.0	2.44-6.67	<0.001
	Female	Ref				
Age group (years)	18-24	Ref				
	25-34	1.78	1.08, 2.94	2.05	1.03, 4.09	0.041
	35-44	2.82	1.73, 4.59	2.59	1.29, 5.21	0.007
	45-59	3.42	2.08, 5.61	3.26	1.59, 6.67	0.001
	≥60	2.30	1.24, 4.27	1.58	0.67, 3.75	0.295
Marital status	Single	Ref				
	Married	1.32	0.93, 1.87	1.27	0.75, 2.15	0.374
	Divorced/widowed	0.62	0.31, 1.23	0.63	0.24, 1.64	0.345
Education	Non-literate	Ref				
	No formal education but read and write	2.11	1.52, 2.92	1.34	0.88, 2.04	0.177
	Primary education	1.42	1.01, 2.00	0.98	0.61, 1.59	0.947
	Secondary and above	1.42	0.86, 2.32	0.95	0.45, 2.00	0.886
Residence	Urban	Ref				
	Rural	1.90	1.13, 3.17	1.94	0.90, 4.21	0.092
Ethnicity	Gurage	Ref				
	Others	0.40	0.19, 0.85	0.45	0.19, 1.08	0.073
Occupation	Farmer	Ref				
	Housewife	3.02	2.13, 4.26	1.10	0.65, 1.86	0.735
	Private business	1.32	0.74, 2.34	1.49	0.70, 3.19	0.299
	Daily laborer	2.64	1.44, 4.83	2.00	0.90, 4.47	0.090
	Others	0.92	0.47, 1.81	0.95	0.36, 2.50	0.916
Perceived relative wealth	Low	Ref				
	Average	1.23	0.93, 1.64	1.22	0.84, 1.77	0.300
	High	1.57	1.04, 2.38	1.56	0.91, 2.67	0.104
Number of adverse life events	None	Ref				
	1-2 events	1.72	1.26, 2.35	1.71	1.18, 2.48	0.005
	≥3 events	2.69	1.91, 3.79	2.12	1.36, 3.32	0.001
Social support	Low	Ref				
	Moderate	0.88	0.67, 1.16	0.73	0.52, 1.02	0.068
	High	0.64	0.40, 1.01	0.41	0.23, 0.72	0.002
Severity of psychological distress	No distress	Ref				
	Mild	1.50	1.05, 2.13	1.26	0.80, 2.00	0.315
	Moderate	1.86	1.24, 2.79	1.37	0.80, 2.35	0.257
	Severe	2.47	1.51, 4.06	2.96	1.49, 5.89	0.002

*significant at *P*-value of <0.05

## Discussion

This study is one of the few studies on hazardous alcohol drinking reported from a rural setting in sub-Saharan Africa. We found high level of hazardous alcohol use in this rural community of Sodo district which is located in the Ethiopian central highlands where consumption of home brewed alcoholic beverages is common.

Previous studies on alcohol in Ethiopia mainly focused on people living in cities which generally reported lower rates of hazardous drinking using CAGE as a screening tool. For instance, the prevalence of hazardous drinking in Addis Ababa was reported to be 2.7 % [12]. Other alcohol studies in rural Ethiopia involved a population of isolated islanders, located at central rift valley lakes, and semi-nomadic populations in Southern Ethiopia. The lifetime prevalence of alcohol dependence in these two areas was 1.5 % and 1.6 % respectively [9, 13]. The report from the neighboring district of Butajira by Alem et al. was 3.7 % [11], slightly higher than Addis Ababa but much lower than Sodo. One explanation for such huge difference could be that residents in Butajira predominantly follow Islam while those in the Sodo district follow the Coptic Orthodox Christian faith which generally tolerates some consumption of alcohol. The other explanation for the difference could be the types of psychoactive substances commonly consumed in the two districts. In Butajira, 50 % of adults were found to be current chewers khat [6, 12]; whereas, khat chewing is much less common in Sodo. Production of local alcoholic beverages is an important income earner for women in Sodo as well which makes it readily available for consumers. The rate of alcohol consumption in Sodo is generally believed to be somewhat similar to other nearby Christian highlanders; the central and northern highlands are predominantly populated by Christians. The other likely reason for the difference in prevalence is the measurement instruments that were used. CAGE generally picks up severe alcohol use disorder compared to FAST which was developed to assess hazardous drinking. A study done in four cities in the UK comparing CAGE with FAST found a sensitivity of 40 % and 93 % for picking hazardous drinking, respectively [21].

The finding from Sodo is the highest prevalence of hazardous alcohol use in Ethiopia reported thus far. Ethiopia has experienced economic boom in recent years which resulted in poverty reduction and economic improvement (www.worldbank.org/en/country/ethiopia). This has resulted in a substantial influx of foreign direct investment into the country. The largest investment from Europe into the country came from big breweries (www.thereporterethiopia.com/index.php/interview/item/1963-its-ethiopias-time). Currently, there is huge expansion of the beer industry with extensive promotion activities going on throughout the country. It is likely to reach a stage of an ‘industrial epidemic’ soon [30]. Although locally brewed alcoholic beverages are predominantly consumed in rural areas, manufactured beverages are increasingly gaining popularity. Nowadays, drinking manufactured liquor and beer is considered a status symbol. Hence, the findings in Sodo are pertinent to the rest of the country.

In this study, hazardous use of alcohol was also found to be associated with being male, age below 60 years, having experienced one or more life events and severe psychological distress. Substance abuse is generally more common in men than women, it is even more so in men living in sub-Saharan Africa where one would find much more abstaining women than men [4, 31]. Although high rates of hazardous alcohol use is common in young people, usually in the form of binge drinking, this phenomenon was found to be less common in Africa compared to western countries [31, 32]. In this study, we found that the rate of hazardous alcohol use increased with increasing age till age 60. Problems arising from excessive alcohol drinking are many; it is an important risk factor for many of the chronic diseases including neuropsychiatric disorders and considered to be among the top causes of global burden of disease, disability and premature death [33]. The presence of severe psychological distress was significantly associated with hazardous alcohol use in this study. This is consistent with studies reported from elsewhere. Although it is difficult to determine causality, it is common to find people who have underlying psychological problems to be more prone to drinking large quantities of alcohol possibly as self medication, or the hazardous use of alcohol may predispose users to psychological distress [34, 35]. Similarly, experiencing one or more adverse life events was also associated with hazardous alcohol use which was consistent with other findings [36].

Limitations in this study include possible underreporting by participants, but it is generally believed that people who drink alcohol report their alcohol intake in a reliable way [37]. The validity of such reports had been confirmed both in community surveys as well as in people who have alcohol dependence [37, 38]. There could also be recall bias or difficulty understanding some of the items as well as issues with translation of the questions as the FAST instrument was not validated in Ethiopia. The possibility of over-reporting due to social desirability or misunderstanding of the questions cannot be ruled out. Measurement bias is also another limitation when using an instrument which is not validated for use in a particular setting. Generally, the FAST instrument was validated as a quick screening instrument for hazardous alcohol use in emergency medical settings elsewhere and has been rarely used in community surveys.

## Conclusion

In summary, the high rate of hazardous alcohol use that was found in this rural Ethiopian primary care setting warrants attention. Moreover, it affects productive members of the society which will have significant impact on families and the country as whole. Public health measures that aim at reducing hazardous alcohol use coupled with a stepped care approach for alcohol use disorder i.e. screening all adults presenting at primary care, brief intervention for hazardous drinking and referral of severe cases of alcohol dependence is recommended.
